# Dynamics of Hepatitis B Virus Covalently Closed Circular DNA: A Mini-Review

**DOI:** 10.3390/microorganisms11030600

**Published:** 2023-02-27

**Authors:** Jie-Li Hu, Ai-Long Huang

**Affiliations:** Key Laboratory of Molecular Biology on Infectious Diseases, Ministry of Education, Chongqing Medical University, Chongqing 400016, China

**Keywords:** hepatitis B virus, cccDNA, half-life, nucleot(s)ide analogues, hepatocyte, turnover

## Abstract

Eradication of cccDNA is an ideal goal of chronic hepatitis B (CHB) therapy. Understanding the changes in the cccDNA pool during therapy provides a basis for developing CHB treatment strategies. On the other hand, the shift in the balance of the cccDNA pool following therapies allowed researchers to investigate the dynamics of cccDNA. Central to the description of cccDNA dynamics is a parameter called cccDNA half-life. CccDNA half-life is not an intrinsic property of cccDNA molecules, but a description of an observed phenomenon characterized by cccDNA pool decline. Since cccDNA has to be in the nuclei of host cells to function, the half-life of cccDNA is determined by the state and destiny of the host cells. The major factors that drive cccDNA decay include noncytopathic effects and hepatocyte turnover (death and division). In some cases, the determining factor is not the half-life of cccDNA itself, but rather the half-life of the hepatocyte. The main purpose of this review is to analyze the major factors affecting cccDNA half-life and determine the areas requiring further study. In addition, the discrepancy in cccDNA half-life between short-term and long-term nucleot(s)ide analog (NUC) therapy was reported. Hypotheses were proposed to explain the multi-phasic decline of cccDNA during NUC therapy, and a framework based on cccDNA dynamics was suggested for the consideration of various anti-HBV strategies.

## 1. Introduction

**Figure 1:**
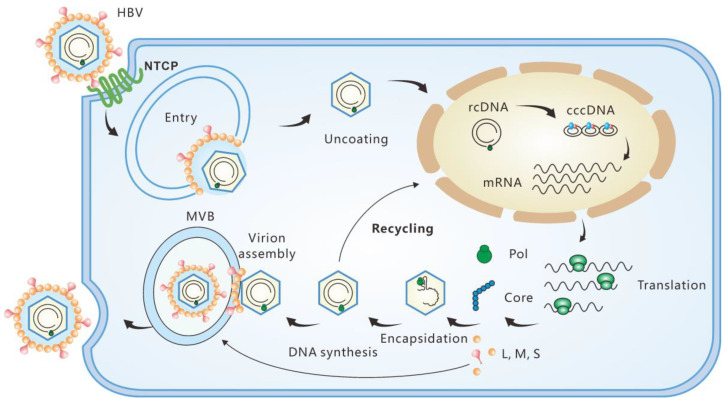
Replication cycle of HBV. The viruses infect hepatocytes by interacting with the receptor sodium taurocholate cotransporting polypeptide (NTCP). Relaxed circular DNA (rcDNA) is released into the nuclei after nucleocapsid uncoating. With the help of the host factors, rcDNA is converted into cccDNA, which serves as the template of viral RNA transcription. The productive nucleocapsid containing progeny rcDNA either secretes through the multivesicular body (MVB) pathway or recycles to the nuclei.

Covalently closed circular DNA (cccDNA) is the first viral product after hepatitis B virus (HBV) infection of hepatocytes. It serves as a stable repository of HBV genetic information in liver cells and represents the most impenetrable barrier to a functional cure for chronic hepatitis B (CHB) [1,2,3]. CccDNA is converted from its precursor, relaxed circular DNA (rcDNA) (Figure 1). With great effort, in almost two decades, researchers have largely characterized the host factors required for cccDNA formation, intermediates formed during the conversion and the functional regulation mechanisms of cccDNA minichromosomes (see reviews [4,5,6]). There is a consensus that the functional cure of CHB requires eradication or persistent suppression of cccDNA [2,7,8,9,10]. However, current therapies based on the nucleot(s)ide analog (NUC) or/and pegylated-interferon-α (PEG-IFNα) have limited efficacy in achieving a functional cure for CHB, necessitating the development of new therapeutic strategies [11,12,13,14,15,16].

It is well known that serum HBV virions are in a highly dynamic equilibrium; approximately 10^11^ viruses decay and are produced daily [17]. Similarly, the cccDNA pool in hepatocytes is in a dynamic equilibrium [18]. Since any treatment to cure CHB must reduce the number of cccDNA molecules in the livers, it is important to understand the dynamic changes in cccDNA quantity during therapy. In fact, cccDNA dynamics can only be revealed by treatments that shift the equilibrium in the cccDNA pool. Both NUC and PEG-IFNα can reduce cccDNA levels either indirectly by suppressing cccDNA synthesis or directly by degrading cccDNA. The decline of cccDNA during therapy denotes a shift in the dynamic balance between cccDNA synthesis and decay. The dynamics of the cccDNA pool can be investigated by exploring this shift. However, cccDNA dynamics have not been extensively discussed to date. In this mini-review, we focused on the property of cccDNA half-life, a critical parameter to describe the dynamics of cccDNA, and the factors that affect it. In addition, the discrepancy in cccDNA half-life between short-term and long-term nucleot(s)ide analog (NUC) therapy was analyzed, and a framework based on cccDNA dynamics was suggested for the consideration of various anti-HBV strategies.

## 2. The Term ‘cccDNA Half-Life’ Does Not Refer to an Intrinsic Property of cccDNA

The half-life of pure cccDNA independent of its host cells is practically meaningless, since cccDNA has to be in the nuclei of host cells in order to function. It is the destiny and environment of the host cells that govern the ‘half-life’ of cccDNA. The cccDNA disappear when the host cells die, and decrease when the host cells divide. CccDNA can also be degraded in noncytopathic ways. The cccDNA pool may exhibit very different half-lives under different conditions. In chimpanzees recovering from an acute HBV infection, cccDNA can decline with a half-life as short as 3 days [19]. In contrast, patients receiving long-term NUC therapy exhibited a very slow cccDNA decline, with a half-life of as long as 26 months [20]. Apparently, ‘cccDNA half-life’ involves the quantified amplitudes of the cccDNA decline phenomenon, which vary considerably depending on the situation. Therefore, this term does not refer to the intrinsic nature of cccDNA, and that the values represent the observed kinetics of cccDNA decay as a pool that would change depending on the circumstances. In this sense, the term ‘apparent cccDNA half-life’, as proposed by Boettler et al., is more accurate than ‘cccDNA half-life’ [18].

## 3. Major Factors That Affect cccDNA Half-Life

Given that cccDNA is located in the nuclei of the host cells and performs its functions, the state of the host cells will exert a significant influence on the dynamics of cccDNA. Therefore, factors affecting the situations of host cells affect cccDNA half-life. Noncytopathic effects and hepatocyte turnover are the major factors impacting cccDNA half-life.

### 3.1. Noncytopathic Effects Contribute to cccDNA Degradation in Acute Infection

Chisari et al. first reported that HBV-specific cytotoxic T lymphocytes could abolish HBV gene expression and replication in the liver of transgenic mice via noncytopathic cytokines [21]. Based on these findings, they postulated that this antiviral process might be primarily responsible for the viral clearance during human HBV infection rather than the destruction of infected cells. To test this hypothesis, they observed the clearance of HBV in acutely infected chimpanzees. In these animals, a significant decrease in HBV replicative intermediates and cccDNA from the liver was observed long before the peak of T cell infiltration and most of the liver disease. Xia et al. also found that HBV-specific T cells inhibit HBV replication and reduce cccDNA in infected cells without the direct contact required for cytolysis [22]. The findings support the presence of a noncytopathic mechanism contributing to the clearance of the virus [23]. Mathematical modeling was later performed to evaluate the extent to which cytopathic and noncytopathic T cell effector functions contribute to the resolution of HBV infection in three acutely infected chimpanzees. CccDNA demonstrated a rapid decay in the first phase of the decline, with a half-life of 3 days. If cell death and cell division were the only mechanisms responsible for clearance of HBV infection, such a short half-life would imply the destruction and regeneration of approximately 11 livers. In contrast, simulation incorporating the cytokine effects indicated significantly less hepatocyte death and regeneration (1.4–2.8 livers) [19]. Studies using woodchuck models also supported that both noncytopathic effects and hepatocyte death were responsible for the elimination of cccDNA during recovery from transient infections [24]. Cytokines associated with this noncytopathic effect include interferon-gamma (IFNγ), tumor necrosis factor-alpha (TNFα), and IFNα/β [22,25]. Later studies showed that IFNα accelerates the decay of DHBV cccDNA in culture [26] and induces the specific degradation of the nuclear HBV cccDNA without hepatotoxicity by up-regulating APOBEC3A and APOBEC3B cytidine deaminases [22,27].

### 3.2. Hepatocyte Turnover Is a Major Factor Promoting cccDNA Decay

The liver is a solid organ with a high regenerative capacity to ensure that the liver-to-bodyweight ratio is always at 100% of what is required for body homeostasis [28]. Hepatocyte turnover is regulated by two closely related events, namely hepatocyte death and division. As hepatocytes die, the number of hepatocytes must be replenished to maintain liver mass and new hepatocytes are produced. Typically, a cell death results in a new cell being produced, and vice versa. It is believed that cccDNA is degraded or eliminated when the host cell dies and cannot infect another hepatocyte.

#### 3.2.1. Evidence That Hepatocyte Turnover Promotes cccDNA Decay

The influence of hepatocyte turnover on cccDNA levels (or infected hepatocytes) was first demonstrated by Mason et al. [29]. An NUC, 2′-deoxycarbocyclic guanosine (2′-CDG), was found to significantly reduce DHBV cccDNA levels (with a half-life of approximately 2 weeks) in the livers of ducks congenitally infected with DHBV. 5-bromo-2’-deoxyuridine (BrdU) labelling showed that hepatocytes proliferation increased by 10-fold after 2 weeks of therapy. This result suggests that the inhibition of viral replication and acceleration of hepatocyte turnover with 2′-CDG therapy caused a rapid clearance of infected hepatocytes. In contrast, 5-fluoro-2’,3’-dideoxy-3’-thiacytidine (524 W), another potent inhibitor of DHBV DNA synthesis that did not expedite the turnover of hepatocytes in ducks, led to a strong inhibition of virus production but a slower rate of decline in the number of infected hepatocytes and cccDNA (with a half-life of more than 6 weeks) than that with 2′-CDG. Addison et al. analyzed the decline of DHBV cccDNA in ducks treated with a combination of lamivudine (LAM) and a dideoxyguanosine prodrug for 5 months [30]. The cccDNA pools in three ducks demonstrated an exponential decline, with half-lives ranging from 35 to 57 days. Liver sections stained with the cell division marker, proliferating cell nuclear antigen (PCNA), showed that animals with a continuous cccDNA loss had a significantly higher number of PCNA-positive nuclei than those whose cccDNA levels had plateaued.

Mason et al. also treated WHV-infected primary hepatocyte cultures with [1-(2-fluoro-5-methyl-β-L-arabinofuranosyl) uracil] (L-FMAU), a reverse transcriptase inhibitor. They found that although L-FMAU caused a 200-fold suppression in viral DNA replication, no significant loss in cccDNA was observed in the infected hepatocytes during the 40-day treatment [31]. In chronically infected woodchucks, L-FMAU treatment for 30 weeks reduced cccDNA levels to between 1.2 and 5.4% of pretreatment levels, suggesting a half-life of 33 to 50 days. This reduction could be explained by an infected-cell death rate of 1.3 to 2.1% per day, which is in reasonable agreement with the PCNA staining results.

Outstanding examples exhibiting the influence of hepatocyte death on cccDNA is from the experimental therapy of HBV infection by T cells that were engineered to express HBV-specific chimeric antigen receptors (CARs) or T cell receptors (TCRs). Protzer’s lab reported that expression of CARs directed against the HBV surface proteins enables human T cells to kill HBV-infected human hepatocytes and to eliminate viral cccDNA in vitro and in vivo [32,33]. T cells stably expressing high-affinity HBV envelope- or core-specific TCRs also effectively control HBV infection in humanized mice by specifically clearing infected hepatocytes without damaging noninfected cells [34].

#### 3.2.2. Effect of Cell Division on cccDNA

In nonproliferating primary cultures of woodchuck hepatocyte infected with woodchuck hepatitis virus (WHV), treatment of L-FMAU did not facilitate loss of cccDNA. No significant loss of cccDNA from the treated cultures was seen between days 8 and 40 post-infection, indicating a cccDNA half-life of at least 32 days [31]. Ko et al. tested cccDNA half-life in an HBV-infected cell line (HepG2-NTCP-K7) in the presence of 2.5% DMSO, which induces cell cycle arrest. Treating cells with entecavir (ETV) reduced cccDNA levels by 48% at day 45 post-infection, suggesting a cccDNA half-life of approximately 40 days in these nondividing cells [35].

During hepatocyte division, the HBV cccDNA minichromosome could be distributed unequally or even lost during mitosis since it is not a cellular chromosome equipped with centromere structures. Chong et al. examined the dynamic changes in HBV cccDNA in different cellular growth stages of a stably HBV-producing cell line, 1.3ES2 [36]. They found that the amount of cccDNA decreased dramatically in the cells during their exponential proliferation, and cccDNA could be removed when proliferating cells were subjected to long term of lamivudine (3TC) treatment. The half-life of cccDNA in the exponentially proliferating cells was approximately 5 days. Once the cells had grown to confluence, the half-life of cccDNA was approximately 9 days, but 20% of the remaining cccDNA was retained stably inside the cells for more than 30 days [36]. To quantify the impact of cell division on cccDNA loss, Tu et al. passaged cells every 3 days to induce mitosis of HBV-infected HepG2-NTCP and HepaRG-NTCP cells. Then, the number of cccDNA copies was measured by precise PCR assays and the number of HBV-expressing cells was monitored over time using reporter viruses. They observed that cccDNA levels undergo a 5-fold decrease after each round of mitosis, which is the exact rate predicted by mathematical models assuming a complete loss of cccDNA in daughter cells [37].

In contrast, some other studies did not observe significant cccDNA loss during cell division. Li et al. determined the distribution of HBV cccDNA in continuously passaged HepAD38 cells using a fluorescence imaging in situ hybridization (FISH)-based assay. The findings showed that nuclear HBV DNA symmetrically distribute to daughter cells. It is worth noting that the detected nuclear DNA was not necessarily cccDNA, since the probes used were not cccDNA-specific [38]. Dandri et al. evaluate whether nuclear cccDNA becomes unstable during cell division in cultures of primary hepatocytes isolated from a WHV chronically infected woodchuck [39]. The cells were treated with epidermal growth factor (EGF) for 24 days to induce proliferation in the presence of adefovir (ADV). They found that cccDNA signals had the same intensity in ADV-treated plates despite the fact that a 50% increase in cell number (70% vs. 105%) did occur when EGF was added. Cautions should be taken while interpreting this result, because the cells seemed to proliferate at a marginal rate during a long period.

To determine the effect of cell proliferation on cccDNA in vivo, Lutgehetmann et al. transplanted primary tupaia hepatocytes (PTHs) chronically infected with woolly monkey HBV (WM-HBV) from chimeric mice into the urokinase-type plasminogen activator (uPA)/severe combined immunodeficiency (SCID)/beige (USB) mouse model. Transplantation of WM-HBV-infected hepatocytes led to an average of 3.8 PTH doublings within 80 days. Remarkably, a median 2-log decline of cccDNA per cell determined during PTH proliferation was due to both dilution of the cccDNA pool among daughter cells and a 0.5-log loss of intrahepatic cccDNA loads [40]. The effect of hepatocyte division on the HBV cccDNA pool was also observed in humanized mice. Primary human hepatocytes (PHH) from HBV-infected humanized mice were serially transplanted into naïve recipients. The findings demonstrated that human hepatocyte division, triggers substantial cccDNA loss even without the involvement of cytolytic mechanisms. CccDNA copies per PHH decreased by 2.4 log during cell division (from day 3 post-transplantation until day 30), and the total cccDNA amounts per liver reduced by 0.7 log during this period [41]. In contrast, Hayashi et al. demonstrated that in HBV-infected human-liver-chimeric mice (PXB-mice), the total cccDNA content did not change during liver repopulation after entecavir treatment. An explanation for the inconsistence is that the experimental procedure used by Hayashi et al. is different from that used by Allweiss et al. In PXB-mice, observation started 8 weeks after transplantation and 4 weeks after HBV infection. Allweiss et al. adopted a different procedure in which human liver cells from USB mice already infected with HBV were transplanted to naïve recipients and observation started 3 days after transplantation. Probably, this procedure maximized the proliferation rate of the cells and thus presented its effect on cccDNA more readily.

#### 3.2.3. Hepatocyte Turnover Rate

**Figure 2:**
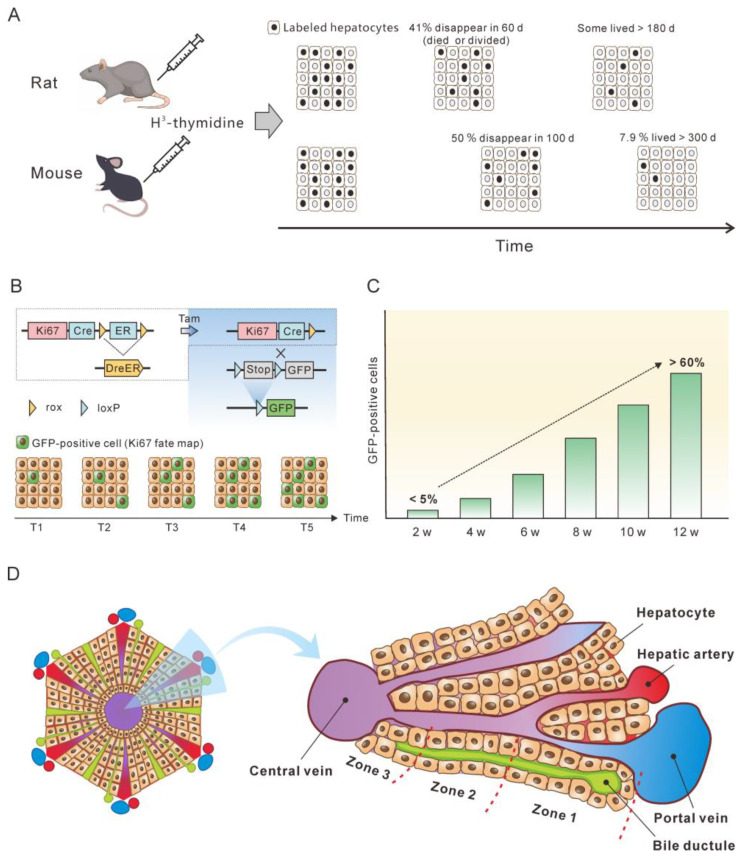
Monitoring the lifespan and proliferation of hepatocytes. (**A**) H^3^-thymidine was injected into rat and mouse models to label hepatocytes and monitor the changes in the labeled cells [42,43]. (**B**) Ki67 expression induced a constitutive expression of GFP which was used to monitor the fate of liver cells. (**C**) From week 2 to week 12, the number of hepatocytes with Ki67-expression-induced GFP expression increased to more than 50%, which represent newly produced hepatocytes. The figure is a modification of a figure from reference [45]. (**D**) Structure of the liver lobule. Hepatocytes in the liver lobule are organized into three zones and those in zones 2 and 1 have higher proliferation rates than those in zone 3 during homeostasis.

Knowing the lifespan or turnover rate of hepatocytes is essential in exploring the dynamics of the cccDNA pool due to the close relationship between cell death and the change in the cccDNA pool. In 1961, Richard determined the lifespan of rat liver cells by tracing tritiated thymidine (H^3^-thymidine) incorporated into cell nuclei [42]. He found that 0.22% of hepatic nuclei were labelled following a single injection of H^3^-thy-midine in normal adult rats, suggesting that a liver would renew in 450 days. Of note, it was estimated that in normal liver approximately 41% of labeled cells had died or divided by 60 days, and the remaining labeled cells had lifespans varying up to at least 6 additional months [42] (Figure 2A). This implies a great heterogeneity of hepatocytes lifespan. For fatty and cirrhotic liver, the lifespan of hepatocytes was considerably less than that of normal liver (26 days vs. 450 days). Again, those hepatocytes exhibited heterogeneity in lifespan. Although 98% of labelled hepatocytes died or divided in 60 days, the remaining 2% of cells did not go further change for at least several months. Magami et al. used H^3^-thymidine to trace the life of hepatocytes in mice [43]. They found that the proportion of labelled hepatocytes decreased from 33.7% to 16.6%, 10.9% and 7.9% after 100, 200 and 300 days (Figure 2A). This indicates an overall hepatocyte half-life of approximately 100 days, but the heterogeneity is apparent at individual cell level, given that 7.9% of the cells survived longer than 300 days [43].

The concept of the balance between hepatocyte death and production provides a basis for estimating hepatocyte death rate by monitoring newly produced hepatocytes over time. Recently, He et al. assessed hepatocyte proliferation in a mouse model using Ki67-induced expression of the fluorescent protein [44]. Their dual recombinase-based method, called Protracer (proliferation tracer, Figure 2B), enabled the continuous recording of the proliferation events of entire cell populations over time in multiple organs, including the liver. Although their primary goal was to characterize the cell source of hepatocytes during homeostasis and regeneration, the study provided valuable information to estimate the turnover of hepatocytes. One set of data revealed that from week 2 to week 12 after tamoxifen induction, a gradual increase in the number of newly produced hepatocytes was observed, as demonstrated by the GFP expression in the cells induced by Ki67 expression. At week 12, over 60% of the total population were GFP-positive cells (Figure 2C). This implies that more than 60% of the hepatocytes died during the course of the 10 weeks, as approximately 60% of the hepatocytes were newly produced at this time. Given that some GFP-positive hepatocytes must have already died, 10 weeks may be a low estimate of the half-life of mouse hepatocytes in homeostasis. This estimate closes to the half-life of hepatocytes (100 days) inferred from the H^3^-thymidine labelling experiments. In addition, different regions of the liver lobule contribute differentially to hepatocyte turnover, and zone 2 is the primary source of new hepatocytes during homeostasis and regeneration (Figure 2D) [44,45].

The half-life of normal human hepatocytes does not permit experimental determination in vivo by lineage or H^3^-thymidine tracing. However, indirect estimation of normal human hepatocyte turnover can be made by immunohistochemical detection of PCNA. PCNA is present in the cell nuclei throughout the cell cycle but binds tightly to chromatin at the peak of the S-phase. Immunofluorescent studies have shown that in cells only PCNA associated with DNA replication sites (S-phase-specific PCNA) can be detected. In contrast, non-S-phase nuclear PCNA, which is present in lower amounts and is not physically associated with DNA replication sites, is likely lost or undetectable by conventional immunocytochemical methods [46,47,48]. In methanol-fixed normal human liver, the PCNA-labeling index was from 0.05% to 0.78% for hepatocytes [49,50]. This translates to 0.15 to 2.34% of hepatocyte death per day, assuming that S-phase lasts for approximately 8 h and that PCNA staining therefore only reflects approximately one-third of the cells in S-phase over a 24 h period [51]. Another proliferation marker, Ki67, can also be used to estimate hepatocyte turnover rate. Farinati et al. found that 0.2% of the hepatocytes were Ki6-positive in the periportal area (zone 1) in the samples from patients with hepatitis B, translating to a 0.6% of hepatocyte death per day. However, this might be a low estimate since the majority of proliferating hepatocytes were found in zone 2 of mouse lobules [44,45].

In summary, normal hepatocytes of rat and mice exhibit a heterogeneity in half-life. The majority of hepatocytes have a half-life of 50–100 days, but a minority of hepatocytes can live longer than 300 days. The death rate of normal human hepatocytes is estimated to be approximately 0.5% per day, equivalent to a half-life of 100 days. However, details of half-life heterogeneity of human hepatocytes need further investigation.

## 4. HBV Genome Recycling and cccDNA Replenishment

Hepatoma cell lines, such as HepG2, transfected stably or transiently with replication-capable HBV genome support cccDNA formation [52,53,54]. These cccDNA must be completely converted from intracellular recycling rcDNA, since these cell lines do not support de novo HBV infection. In a HepG2 cell line expressing sodium taurocholate cotransporting polypeptide (NTCP) (HepG2-NTCP-K7), transduction of L-HBsAg-deficient, but replication-competent overlength HBV genome (HBV1.3L-) via an adenoviral vector (Ad-HBV1.3L-) also resulted in cccDNA. Because cccDNA formation in this system can only result from nuclear import of capsids, this demonstrates that the intracellular recycling pathway of rcDNA-containing capsids is a driver of cccDNA replenishment [35]. Recently, Kostyushev et al. found that CRISPR-Cas9-mediated inactivation of HBV cccDNA is not sufficient for curing infection, because HBV replication rapidly rebounds in their system due to formation of HBV cccDNA from recycling rcDNA [55].

However, in cells supporting HBV infection, intracellular recycling of rcDNA seems not to play a primary role in establishing and sustaining the cccDNA pool. In HepG2-NTCP A3 cells infected with wild-type HBV or HBV that do not encode core protein (ΔHBc HBV) from its genome, cccDNA levels were comparable over 9 weeks of infection [56]. ΔHBc HBV do not produce rcDNA after infection because of no core protein, suggesting that intracellular recycling of rcDNA is not critical in sustaining the cccDNA pool in this model. Volz et al. treated humanized uPA/SCID mice infected with HBV with the entry inhibitor, myrcludex B (or bulevirtide), from week 3 to week 9 post-infection. They found that intrahepatic cccDNA loads did not differ significantly between these mice and mice that were sacrificed 3 weeks post-infection [57]. This result indicates that intracellular recycling of rcDNA does not increase the amount of cccDNA in those cells in which infection has already been established. Possibly, there is a mechanism for HBV to limit cccDNA in a low copy number in primary human hepatocytes [52]. This number might be easily fulfilled by de novo infection and maintained thereafter in quiescent cells, letting it unnecessary to recycle rcDNA. Probably, recycling acts as a ‘salvage’ pathway to rescue an occasional loss of cccDNA (e.g., by the activity of nucleases) rather than a strong ‘driver’ for counteracting ongoing degradation [56].

## 5. cccDNA Decay Dominates the Second Phase of Serum HBV Decline during Short-Term NUC Therapy

A two-phase decline in serum HBV has repeatedly been observed during short-term NUC therapy. Tsiang et al. reported a biphasic clearance of serum HBV in a cohort of 10 chronic hepatitis B patients receiving adefovir (ADV) for 12 weeks [58]. The initial, fast phase of viral load decline reflects the clearance of HBV particles from the serum, with an average half-life of 1.1 days. The second, slower phase of viral load decline has a mean half-life of 18 days and is believed to closely mirror the rate-limiting process of infected cell loss. Typically, the two phases’ inflection points were found between weeks 1 and 2. Wolters et al. also recorded a biphasic viral load decline in 10 CHB patients receiving entecavir (ETV) for 28 days. The half-life of serum HBV decline in the first phase was 16 h, and 10.7 days in the second phase [59], with inflection points occurring within 1 week of therapy. The bi-phasic decline of serum HBV was also reported in patients receiving core protein allosteric modulators. In a cohort of 35 patients treated with RG7907 for 28 days, HBV DNA declined fast in the first phase showing a half-life of 17 ± 6 h, and declined slowly in the second phase with a half-life of 6 ± 0.8 days [60].

The decay of the cccDNA pool is the most probable mechanism for the second-phase viral decline, since a model without cccDNA decay would predict a viral load plateau instead. Assuming that the cccDNA pool remained constant during therapy, this pool would produce an equal amount of new viruses at any time point, but at a slower rate (inhibiting efficiency) than in the natural steady state. For example, if a patient had a total serum virus load of 2 × 10^11^ and thus produced 10^11^ viruses per day before therapy (equal to the number of viruses lost per day), 10^9^ viruses would be produced daily under a 99% inhibiting efficiency during therapy. Serum HBV levels enter the fast decline phase first, declining to 2 × 10^9^ after approximately 7 days (7 half-lives). Assuming a constant cccDNA pool, 10^9^ viruses would also be produced at this time. This would be in accordance with the amount of serum HBV that was eliminated each day (half of 2 × 10^9^), predicting that the serum viral load would not decrease further. However, the experimental observations contradicted this hypothesis. Of note, a different inhibiting efficiency could only affect the first phase. For example, it would take 3.3 days for an agent with a 90% inhibiting efficiency (allowing the production of 10^10^ new viruses per day) to reduce the total viral load from 2 × 10^11^ to 2 × 10^10^, after which it would plateau.

**Figure 3:**
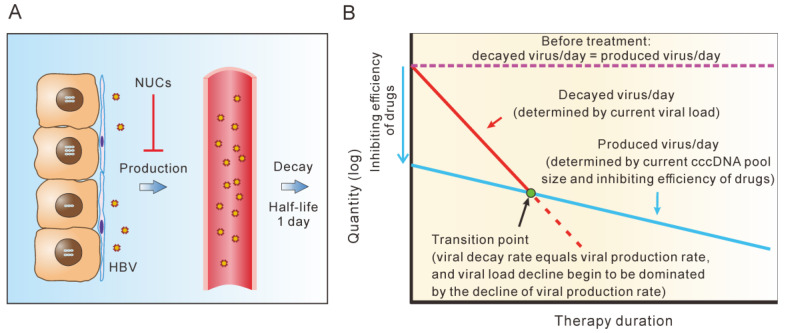
cccDNA decline is responsible for the second phase of serum viral load decrease during NUC therapy. (**A**) Serum HBV has a half-life of one day [17], and new viruses derived from the transcription of cccDNA replenish serum viral load during homeostasis prior to treatment. NUC treatment alters the balances in serum viral load and cccDNA pool size by inhibiting the production of new viruses [58]. (**B**) The serum HBV decay rate (amount of viruses that decay each day) is determined by the current viral load, and the production rate of new viruses is determined by the cccDNA pool size. Both rates decrease over the course of NUC treatment because the total viral load and the cccDNA pool size both decline. However, the decay rate declines faster than the production rate since serum HBV has a much shorter half-life than cccDNA (or infected hepatocyte). At the transition point, the viral decay rate equals the production rate. Subsequently, the production rate (or cccDNA) shows a faster decline than the serum viral load.

Therefore, decreases in cccDNA or hepatocyte turnover should be involved in the second phase of decline in serum viral load during short-term therapy of NUC. The first, fast phase of viral load decline results from the short half-life of serum HBV (1 day). The subsequent decline in serum viral load must be primarily caused by the decline of cccDNA due to the daily production of new viruses approaching the daily decay of viruses. As depicted in Figure 3, the amount of serum virus decayed per day (decay rate), which is determined by the current serum viral load, decreases with time passes. However, the daily amount of newly produced virus also declines during NUC treatment, which is determined by the cccDNA pool size and inhibiting potency of the drugs used. The daily decay of virus declines faster than the daily production of new virus. Before the transition point, the decline of serum viral load is primarily determined by the decrease in serum virus levels. After the transition point, the amount of decayed viruses per day is roughly equal to the number of newly produced viruses. Hence, the viral load is primarily determined by the decline in production rate.

Assuming that the decline of cccDNA is the primary cause of the second phase of decline in serum viral load, the cccDNA half-life would correspond to approximately 6–18 days [58,59,60,61] (Table 1). This value is comparable to the result (16 days) obtained by Nowark et al. using a different method [17]. Notably, the researchers appeared to explain the decline in production rate completely by the death of infected cells, and they deduced the half-life of hepatocytes on this basis. We argue that this observation is better to explain this by cccDNA decline, which involves cell death, cell division and noncytopathic effects. The half-lives of infected cells might have been overestimated, perhaps by a factor of less than two.

## 6. A Significant Variation in cccDNA Half-life Was Observed during Different Terms of NUC Therapy

Direct analyses of cccDNA in the liver have also been performed in patients receiving long-term therapy. However, varying cccDNA decline rates have been observed throughout the course of the therapy. Data on cccDNA decline from 10 previous studies were collected (Table 2). These studies provided data on the cccDNA of paired liver biopsies (pre- and post-treatment) from the same patients, allowing the assessment of the cccDNA half-life. After 48 weeks of NUC therapy, cccDNA declined by 0.7 log for LAM therapy (n = 146), 0.8 log for ADV (n = 22) and 0.9 log for ETV (n = 159) or 1.0 log (another study, n = 40). Table 2 also includes studies that used a combination therapy or had a shorter observation period (12 weeks). The average cccDNA decline within 48 weeks is usually less than 1 log, corresponding to a half-life of more than 14 weeks.

A recent study indirectly analyzed cccDNA turnover by monitoring the composition of HBV RNA from different viral quasi-species during NUC therapy [71]. They found that LAM^R^ mutations emerged and increased from undetectable to 40–90% within 16–28 weeks in serum HBV RNA from telbivudine-treated patients experiencing virological breakthrough. From these results, the cccDNA half-life for the majority of patients was inferred to be <12 weeks. However, these cccDNA half-lives might be overestimated (shorter than reality), since the composition of LAM^R^ would be influenced by the sizes of cccDNA pools, which most likely increased during the breakthrough.

**Figure 4:**
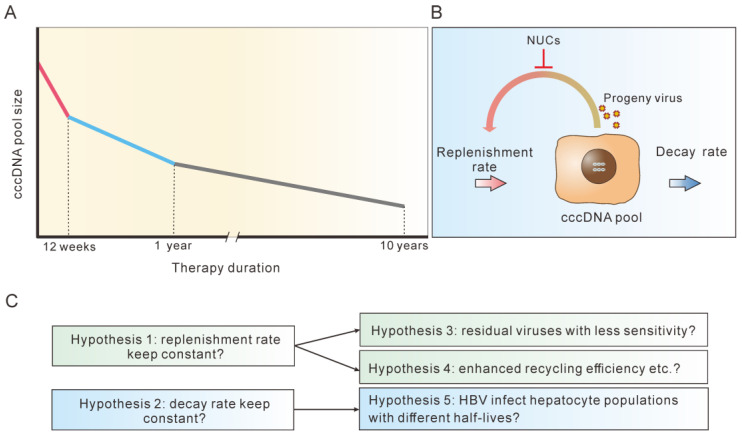
Explanations for the multi-phasic decline of cccDNA during long-term NUC therapy. (**A**) cccDNA declined in a multi-phasic model during long-term NUC therapy ([70], Table 1 and Table 2). (**B**) NUC shifts the balance of the cccDNA pool by reducing replenishment. (**C**) There are several hypotheses for the multi-phasic decline of cccDNA during long-term NUC therapy.

Three studies were performed with a long-term observation period of at least 60 months. One of the three studies used three sequential samples from each patient while they were receiving NUC treatment (baseline, 1 year and a long-term point), which provided useful information for our analysis. This study reported a 1.03 log cccDNA reduction within the first year, equivalent to a half-life of 14.1 weeks, which is close to the half-life calculated above. Within the next 9 years, the same cohort experienced a further 1.94 log cccDNA reduction, which corresponds to a half-life of 76 weeks (this value was an underestimate because the cccDNA levels were below the detection limit in half of the long-term samples). This half-life was approximately 5 times longer than during the first year of treatment. Furthermore, HIV and HBV co-infected patients who received NUC treatment for an average of 65.8 months displayed half-lives of 36 weeks (HBe+) and 112 weeks (HBe−), both of which were significantly longer than the half-life during the first year of therapy. In addition, another study that tracked cccDNA changes during 60 months of NUC treatment reported a 1.56 log cccDNA reduction. Assuming that the cccDNA decreased by 0.8 log in the 1st year, the cccDNA reduction over the next 48 months would be 0.76 log, equivalent to a half-life of 82 weeks. Collectively, these findings demonstrated that during NUC treatment, cccDNA declines with varying half-lives, ranging from 1–2 weeks within the first 12 weeks, to 14 weeks within the first year, to 36–112 weeks within 2–10 years. Collectively, cccDNA declined in a multi-phasic model during long-term NUC therapy (Figure 4A).

## 7. Possible Explanations for the Multi-Phasic Decline of cccDNA during NUC Therapy

At the steady pretreatment state, the daily newly synthesized cccDNA must equal the daily decayed cccDNA in order to maintain the cccDNA pool size (Figure 4B). The rate of new cccDNA formation is mainly determined by the quantity of newly produced viruses and core particles, which lead to new infection or intracellular recycling [35,52]. The cccDNA decay rate primarily depends on the hepatocyte turnover rate and noncytopathic effects that degrade cccDNA. Assuming that during NUC treatment, new virus production is suppressed with a constant efficiency at any given time point (Hypothesis 1), and that the cccDNA decay rate is kept constant (Hypothesis 2), the cccDNA pool will continue to decline and eventually disappear (Figure 4C).

However, the fact that the cccDNA pool size declines in a multi-phasic manner and rarely disappears opposes one or both of the hypotheses. Rejection of Hypothesis 1 implies that the inhibition efficiency of NUC decreases (Hypothesis 3), or the conversion efficiency of newly produced viruses into cccDNA increases with therapy duration (Hypothesis 4). In both circumstances, the efficiency of cccDNA synthesis would be relatively higher in latter stage of therapy than in earlier stage. Hypothesis 3 may be plausible in the case of residual cccDNA producing HBV variants with lower sensitivity to the NUC being used. Comparing the sensitivity of residual variants with that of initial viruses would help to clarify this issue. Hypothesis 4 requires mechanisms such as enhanced intracellular recycling efficiency of HBV DNA-containing capsid particles into nuclei to form cccDNA. However, this possibility seems remote given that intracellular recycling of DNA-containing nucleocapsids is not essential for the maintenance of HBV cccDNA [56].

If Hypothesis 2 is rejected, it would imply that cccDNA decays at different rates depending on the stage of therapy. For example, after a year of therapy, cccDNA would decay more slowly than it would have earlier. This suggests HBV-infected hepatocytes would have different half-lives, since cccDNA half-life is primarily determined by the half-life of hepatocytes (assuming NUC does not affect noncytopathic effects) (Hypothesis 5). In this scenario, cccDNA residing in hepatocytes with a short half-life would be eliminated at a faster rate, leading to a relatively larger slope in the declining curve. In contrast, cccDNA residing in hepatocytes with a long half-life would be eliminated slowly, resulting in a smaller slope. Previous studies indicate that hepatocyte half-life of rat and mouse are heterogenous and a small part of cells lived much longer than other cells [42,43]. Recent studies in mice have confirmed that hepatocytes in distinct liver lobule zones exhibit different proliferation activity [44,45]. Human hepatocytes can also be divided into different clusters according to the transcriptional profiles, as shown by single-cell sequencing [72,73]. Although the turnover rates of these diverse hepatocytes remain unknown, it is possible that a small part of hepatocytes, such as those in rat and mouse, have a long half-life. Given that the proportion of HBV-infected hepatocytes, both in acute and chronic infection, can be or close to 100% in some patients [19,74,75,76,77], viruses have a big chance to infect those long-lived hepatocytes. Testing this and the other four hypotheses would greatly advance our knowledge of the forces controlling the cccDNA dynamics during therapy (Figure 4C).

## 8. Considering CHB Treatments in Light of cccDNA Dynamics

**Figure 5:**
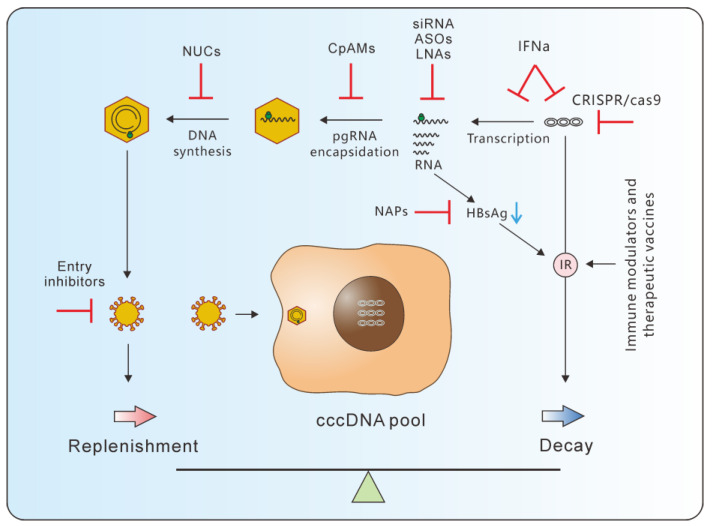
A framework for considering various anti-HBV strategies. Effective therapies shift the balance of the cccDNA pool by either blocking replenishment, accelerating cccDNA decay or both. NUCs, CpAMs, entry inhibitors, siRNA/ASO/LNA and PEG-IFNα all can inhibit cccDNA replenishment indirectly by suppressing steps upstream of cccDNA synthesis. SiRNA/ASO/LNA, NAPs, PEG-IFNα and immune modulators affect cccDNA decay directly or indirectly by facilitating immune restoration.

The balance between cccDNA replenishment (synthesis) and decay during homeostasis provides an alternative framework for considering the effects of various therapies. An effective treatment could either slow down the cccDNA replenishment, speed up cccDNA decay, or exert both effects (Figure 5). NUCs inhibit viral DNA synthesis, which reduces the amount of newly productive viruses and slows the synthesis of downstream cccDNA. Capsid protein assembly modulators (CpAM) interfere with pregenomic RNA (pgRNA) encapsidation into the capsid [78,79,80,81], which also reduces viral production and cccDNA replenishment. Entry inhibitors of various types, including peptides, monoclonal antibodies and compounds [82,83,84], suppress cccDNA replenishment by preventing viruses from entering the cells. None of these treatments affect cccDNA decay, if there are no unintended effects.

SiRNA, anti-sense oligonucleotide (ASO) and locked nucleic acid (LNA) suppress new virus production and cccDNA replenishment by destroying virus RNA directly or suppress cccDNA transcription by reducing HBx level [85,86]. On the other hand, the expression of the surface antigen decreases as a result of preS2/S RNA degradation [85,87,88,89,90]. This may alleviate immune repression brought on by high antigen load and restore the HBV-specific immune response. Indeed, experiments in mouse models demonstrated that siRNA treatment following the administration of a therapeutic vaccine improved HBV-specific immunity and facilitated virus clearance [91]. In clinical trials, an ASO (bepirovirsen or GSK3228836) caused a transient and self-resolved ALT elevation in some patients who experienced a significant HBsAg reduction, indicating that immune reconstruction may occur in these patients [92,93]. Apparently, this immune reconstitution accelerated hepatocyte turnover through cytotoxic effects. Nucleic acid polymers (NAP) can also lower HBsAg load by inhibiting HBsAg release from the cells, and thus might help to restore immune response [94]. PEG-IFNα affects both cccDNA replenishment and decay by simultaneously inhibiting cccDNA transcription and degrading cccDNA [26,27,95]. Endonucleases such as CRISPR/cas9, ZFNs and TALENs affect cccDNA decay by directly cleaving/editing cccDNA molecules [96,97,98]. Immune modulators, such as Toll-like receptor agonists [99,100], PD-L1 antibodies [101,102,103], and therapeutic vaccines [104,105,106,107,108,109], were designed to restore innate immunity or HBV-specific immune response, which is believed to both restrict viral replication and accelerate cccDNA decay.

The cccDNA half-life required for the elimination of cccDNA within a finite course of treatment (e.g., 1 year) can be calculated. Assuming that a liver contains 10^10^ copies of cccDNA in total (approximately 0.1 copy/cell) before treatment, and the patient receives an ideal therapy that can completely block the production of progeny viruses (so that any cccDNA replenishment can be ignored), the cccDNA half-life should be 10 days. This value is within the range of some estimates derived from studies of short-term NUC therapy (Table 1). However, as was mentioned in Section 5, the cccDNA pool does not decline at a constant rate under long-term NUC therapy. This raises another hypothesis (Hypothesis 5), which describes the heterogeneity of the cccDNA pool’s half-life due to the varying half-lives of hepatocyte populations. If this hypothesis is true, a much longer course of treatment should be expected for the complete elimination of cccDNA from the liver, even if high-efficiency agents are used to inhibiting viral production but no effect on cccDNA decay. Under this scenario, a therapy that can affect the cccDNA decay rate is highly desired.

Although it would be ideal for increasing cccDNA decay solely through noncytopathic effects, creating such methods might not be feasible. CRISIPR/cas9 strategies targeting cccDNA were successful in in vitro and animal models [110,111,112]. However, the delivery and editing efficiency and safety issues associated with off-targeting have yet to be solved for clinical usage. IFNα was reported to degrade cccDNA but with many side effects and is effective for a minority of patients. A recent study fused IFNα with a PD-L1 antibody and found the anti-PDL1-IFNα heterodimer preferentially targeted the liver and could overcome HBV-induced immune tolerance to an HBsAg vaccine in a mouse model [113]. This sophisticated approach used the PD-L1 antibody to target IFNα to HBV-infected cells with high PD-L1 expression and block immune checkpoints simultaneously. This approach offers a promising translatable therapeutic strategy for the functional cure of CHB. The first-in-class orally available cccDNA destabilizer, ccc-R08, was recently discovered [114]. Although its clinical use might be problematic because of safety concerns, it provides a proof of concept for developing noncytopathic compounds accelerating cccDNA decay. Strategies that involve initially lowering HBsAg levels first using siRNA, ASO or other compounds, followed by therapeutic vaccine and immune modulators treatment might be worth exploring [91]. If these strategies can restore innate and HBV-specific immunity, a functional cure for CHB would be achievable with an accelerated cccDNA decay rate.

## 9. Conclusions

The genetic materials of HBV, such as HBV DNA, HBV RNA and cccDNA, are distributed in different compartments of the host and exhibit different half-lives. CccDNA in the nucleus decay much slower than viral particles in serum, resulting in a two-phase decline in serum viral load during short-term NUC therapy. Current evidence indicates that cccDNA also declined in a multi-phasic manner during long-term NUC therapy. Considering that cccDNA half-life is mainly determined by the hepatocyte turnover rate in the immune-inactive stage, cccDNA may be distributed across hepatocyte populations with different half-lives. If this hypothesis is true, strategies should be developed to accelerate cccDNA decay in order to reduce cccDNA levels within a set treatment period. Direct antiviral agents (DAA) such as NUC can be used to inhibit cccDNA replenishment, and new agents such as CpAM would support this position even more. However, there is still no efficient treatment promoting cccDNA decay on the market. Strategies are currently being developed to reestablish innate and HBV-specific immune responses.

## Figures and Tables

**Table 1:** cccDNA half-life during short-term NUC therapy.

Therapy	Sample Size	Observation Period	Half-Life of Serum Virion	Half-Life of Infected Cells *	Reference
LAM	45	28 days	1 day	16 days (10–100 days)	[17]
ADV	13	12 weeks	1.1 days	18 days (11–30 days)	[58]
LAM	21	4 weeks	17 h	7 days	[61]
ETV	10	4 weeks	16 h	10.7 days	[59]

* Half-life of infected cells in the original publications should be half-life of cccDNA.

**Table 2:** cccDNA half-life during long-term therapy.

Therapy	Sample Size (Patients)	Observation Period	cccDNA Reduction	cccDNA Half-Life	Reference
ADV	22	48 weeks	0.8 log		[62]
PEG-IFNα-2b + ADV	26	48 weeks	2.4 log		[63]
PEG-IFNα-2a + ADV	40	48 weeks	1.03 log (HBeAg (+)) 0.44 log (HBeAg (−))		[64]
ETV or LAM	305	48 weeks	0.9 log (ETV) 0.7 log (LAM)		[65]
ETV	40	48 weeks	1 log		[66]
NUC	117	52 weeks	0.93 log		[67]
ADV	15	12 weeks	0.65 log		[68]
ETV or ADV	54	60 months	1.56 log		[69]
TDF	27	6.9 years		8.6 months (HBeAg+) 26.2 months (HBeAg−)	[20]
NUC	43	72–145 months		1.03 log in the 1st year, 2.94 log in 2–10 year	[70]

## Data Availability

No new data were used in this manuscript.

## List of Abbreviations

cccDNA, covalently closed circular DNA; HBV, hepatitis B Virus; CHB, chronic hepatitis B; NUC, nucleot(s)ide analogue; PEG-IFNα, pegylated-interferon-α; IFNγ, interferon γ; TNFα, tumor necrosis factor α; IFNα/β, interferon α or β; 2′-CDG, 2′-deoxycarbocyclic guanosine; BrdU, 5-bromo-2’-deoxyuridine; LAM, lamivudine; 524W, 5-fluoro-2’,3’-dideoxy-3’-thiacytidine; PTHs, primary tupaia hepatocytes; L-FMAU, [1-(2-fluoro-5-methyl-β-L-arabinofuranosyl) uracil]; WM-HBV, woolly monkey HBV; uPA, urokinase-type plasminogen activator; SCID, severe combined immunodeficiency; PCNA, proliferating cell nuclear antigen; ADV, adefovir; ETV, entecavir; HBe, HBV e antigen; CpAM, capsid protein assembly modulators; pgRNA, pregenomic RNA; ASO, anti-sense oligonucleotide; LNA, locked nucleic acid; NAP, nucleic acid polymers; ZFNs, zinc finger endonucleases; TALENs, transcription activator-like effector nucleases; PD-L1, programmed death ligand 1.
